# Rotavirus vaccine effectiveness in low-income settings: An evaluation of the test-negative design

**DOI:** 10.1016/j.vaccine.2016.10.077

**Published:** 2017-01-03

**Authors:** Lauren M. Schwartz, M. Elizabeth Halloran, Ali Rowhani-Rahbar, Kathleen M. Neuzil, John C. Victor

**Affiliations:** a Department of Epidemiology, School of Public Health, University of Washington, Seattle, WA, United States; b Department of Biostatistics, School of Public Health, University of Washington, Seattle, WA, United States; c Vaccine and Infectious Diseases Division, Fred Hutchinson Cancer Research Center, Seattle, WA, United States; d Center for Inference and Dynamics of Infectious Diseases, Seattle, WA, United States; e Center for Vaccine Development, University of Maryland School of Medicine, Baltimore, MD, United States; f Center for Vaccine Innovation and Access, PATH, Seattle, WA, United States

**Keywords:** Rotavirus, Vaccine, Test-negative design

## Abstract

**Background:**

The test-negative design (TND), an epidemiologic method currently used to measure rotavirus vaccine (RV) effectiveness, compares the vaccination status of rotavirus-positive cases and rotavirus-negative controls meeting a pre-defined case definition for acute gastroenteritis. Despite the use of this study design in low-income settings, the TND has not been evaluated to measure rotavirus vaccine effectiveness.

**Methods:**

This study builds upon prior methods to evaluate the use of the TND for influenza vaccine using a randomized controlled clinical trial database. Test-negative vaccine effectiveness (VE-TND) estimates were derived from three large randomized placebo-controlled trials (RCTs) of monovalent (RV1) and pentavalent (RV5) rotavirus vaccines in sub-Saharan Africa and Asia. Derived VE-TND estimates were compared to the original RCT vaccine efficacy estimates (VE-RCTs). The core assumption of the TND (i.e., rotavirus vaccine has no effect on rotavirus-negative diarrhea) was also assessed.

**Results:**

TND vaccine effectiveness estimates were nearly equivalent to original RCT vaccine efficacy estimates. Neither RV had a substantial effect on rotavirus-negative diarrhea.

**Conclusions:**

This study supports the TND as an appropriate epidemiologic study design to measure rotavirus vaccine effectiveness in low-income settings.

## Highlights

• Rotavirus vaccines (RVs) have been recently introduced in low-income settings.
• Accurate post-introduction monitoring of RV effectiveness is important.
• The test-negative design (TND) is used to measure RV effectiveness.
• The TND was evaluated using randomized trials of RV in sub-Saharan Africa and Asia.
• The TND is an appropriate method to measure RV effectiveness in low-income settings.

## 1 Introduction

Globally, an estimated 200,000 deaths due to rotavirus diarrhea occur annually in children <5 years old, with a majority of the burden in low-income settings [1]. Starting in 2006, two rotavirus vaccines have been introduced worldwide; GlaxoSmithKline’s live-attenuated human monovalent vaccine (Rotarix [RV1]) and Merck’s live-attenuated pentavalent human-bovine reassortant vaccine (RotaTeq [RV5]). Large multi-site randomized controlled trials (RCTs) of RV1 and RV5 in low-income settings have demonstrated moderate vaccine efficacy against severe rotavirus gastroenteritis in the first year of life (VE: 51–64%) [2], [3], [4], [5], [6]. As of May 1, 2016, rotavirus vaccines have been introduced nationally in 38 Gavi-eligible countries. However, many high-burden countries have not introduced the vaccine and approximately 70% of the world’s infants still do not have access to rotavirus vaccine [7]. Accurate post-introduction monitoring of effectiveness measures is important as results can influence the adoption of rotavirus vaccines in new areas and sustain support in countries where vaccines have been introduced.

Case-control studies are an efficient means to monitor effectiveness and provide confidence in vaccine performance. In low-income settings, identifying community controls, either using a demographic surveillance system or sampling the community in-person, can be impractical and expensive. Hospital controls can be used to minimize bias due to healthcare seeking behavior. However, for rotavirus vaccine studies, careful consideration must be made to use hospital controls without diarrhea or any illness associated with vaccine-preventable diseases. The test-negative design (TND) can theoretically overcome the limitations of both traditionally-used control groups, while also limiting bias due to healthcare seeking behavior [8]. TND rotavirus vaccine studies enroll cases presenting to a medical facility for acute gastroenteritis and are rotavirus-positive using standard laboratory methods. Controls include those presenting to a medical facility with the same pre-defined case definition of acute gastroenteritis, but are rotavirus-negative. Both traditional case-control and test-negative study designs require rotavirus testing on infants presenting to the clinic with diarrhea to identify cases. The TND is efficient and cost-effective in that those testing-negative for rotavirus serve as the control group, instead of being excluded from the study.

The TND has been used extensively to measure annual influenza vaccine effectiveness [8], [9]. Simulation experiments have validated the test-negative design for influenza vaccine under specific core assumptions: (1) vaccine has no effect on the incidence of non-influenza pathogens, (2) a highly sensitive and specific laboratory test is used for pathogen detection, and (3) other sources of bias present in observational studies are minimized [8], [9], [10], [11], [12], [13], [14]. De Serres et al. validated the TND for influenza vaccine utilizing RCT databases to verify the accuracy and precision of TND estimates and to test the assumption that the vaccines had no effect on non-influenza respiratory illness [15]. RCTs are appropriate to validate this design due to limited selection bias and confounding as a result of randomization and blinding, the use of standardized laboratory testing, and enhanced surveillance. Derived test-negative vaccine effectiveness estimates for influenza vaccines were almost identical to the original RCT vaccine efficacy estimates. Importantly, the vaccine coverage in the test-negative controls represented the vaccine coverage in the underlying study population, upholding the key assumption that the vaccine had no effect on non-influenza illness. Together, these results indicated the TND was a valid epidemiologic study design to measure influenza vaccine effectiveness [15].

The TND is being increasingly used to estimate rotavirus vaccine effectiveness in middle- and low-income settings due to its low cost and feasibility,[16], [17], [18], [19], [20], [21], [22], [23], [24], [25], [26] but little has been done to assess this epidemiologic study design in the context of rotavirus vaccine effectiveness in low-income settings. In the present analysis, RCT databases for RV1 and RV5 in sub-Saharan Africa and Asia were used to evaluate the TND.

## 2 Methods

### 2.1 Participants and study design

Databases from three multi-center, double-blind, individual-randomized, placebo-controlled, trials of rotavirus vaccines in sub-Saharan Africa and Asia were used [2], [3], [4], [5], [6]. Table 1 summarizes location, vaccine schedule, per-protocol population size, and surveillance type of the three RCTs.

#### 2.1.1 RV1

This trial was conducted in South Africa and Malawi. Between 2005 and 2007, 4939 healthy infants aged 5–10 weeks were randomly assigned to one of three groups in a 1:1:1 ratio: two doses of RV1, three doses of RV1, or three doses of placebo. Gastroenteritis was defined as three or more loose or watery stools within 24 h. Clinical characteristics of each diarrheal episode were documented to define severity based on the Vesikari score [27]. Stool samples were tested for rotavirus using enzyme-linked immunosorbent assay (ELISA). The primary outcome was at least one episode of severe rotavirus gastroenteritis (Vesikari score ⩾11). Vaccine efficacy was estimated during the period from two weeks after the last dose until the first year of age. Within each study site, a sub-cohort was followed into the second year of life. The mean age at the end of follow-up was 14 months and 19 months for South Africa and Malawi, respectively.

#### 2.1.2 RV5

Two trials of RV5 were conducted in sub-Saharan Africa and Asia between 2007 and 2009. Both trials were conducted under similar protocols; however, the trials were powered and implemented separately. In sub-Saharan Africa, 5468 healthy infants were enrolled in Ghana, Kenya, and Mali. In Asia, 2036 healthy infants were enrolled in Bangladesh and Vietnam. Infants aged 4–12 weeks were randomly assigned to one of two groups in a 1:1 ratio: three doses of RV5 or three doses of placebo. As in the RV1 trial, severe rotavirus gastroenteritis was defined based on a positive ELISA laboratory result and Vesikari score ⩾11. Vaccine efficacy was estimated during the period from two weeks after the last dose until the end of follow-up (March 31, 2009). The mean age at the end of follow-up was 20 months and 19 months for sub-Saharan Africa and Asia, respectively.

For the purposes of this analysis, participants with an episode of severe diarrhea meeting the pre-defined case definition and with an available ELISA test result were categorized as a case if the test was positive for rotavirus or a control if the test was negative for rotavirus. Continuous diarrheal surveillance during the study period allowed for the identification of multiple diarrheal episodes for each participant. A participant was defined as a case if at least one severe rotavirus-positive diarrheal episode occurred during follow-up. A participant was defined as a control if at least one severe rotavirus-negative diarrheal episode occurred during follow-up and the participant had no severe rotavirus-positive episodes.

### 2.2 Statistical analysis

Logistic regression was used to estimate the relative odds and associated 95% confidence intervals (CI) of severe rotavirus-positive diarrhea compared to severe rotavirus-negative diarrhea by vaccine and placebo status. TND vaccine effectiveness (VE-TND) was defined as (1 − Odds Ratio) × 100. The relative percent difference between VE-TND and the original RCT vaccine efficacy (VE-RCT) was calculated. To estimate the influence of vaccine on rotavirus-negative diarrhea (VE-NEG), the relative risk of severe rotavirus-negative diarrhea in the vaccine group compared to the placebo group with exact 95% CIs were calculated. VE-NEG was defined as (1 − Relative Risk) × 100.

The analysis was based on the per-protocol participant populations. The primary analysis of each RCT, combining country-level estimates, was replicated. Additionally, analyses were stratified by country. The RV1 trial was powered to estimate vaccine efficacy for two and three doses separately, therefore analyses were replicated by these dosing combinations. Each trial included diarrheal surveillance on all or a subset of participants into the second year of life. Analyses were conducted for the complete follow-up period (<2 years of age) and separately for diarrheal episodes identified during the first (<1 years of age) and second (1–<2 years of age) years of life. Analyses conducted within the second year of life did not exclude participants with diarrheal episodes during the first year of life. For the RV1 trial, different methods of enrollment into the second year were used for each country; therefore, analyses for the second year of life and complete follow-up were conducted separately for South Africa and Malawi.

In practice, differences in age and time at presentation between cases and test-negative controls are controlled by matching or by adjusting analyses by both month and year of birth and month and year of presentation. Additional analyses were restricted by rotavirus season in Ghana and Mali due to the observed seasonality in these regions. To replicate the primary efficacy results of the RV5 trial, diarrheal episodes identified during the rotavirus seasons in Ghana and Mali were combined with the year-round diarrheal episodes in Kenya to estimate country-combined estimates.

Analyses were completed using STATA version 14 (Stata Corporation, College Station, TX, USA) and the SAS Clinical Trial Data Transparency software system through the online GSK portal (SAS Institute Inc., Cary, NC, USA).

## 3 Results

Table 2 shows the derived test-negative vaccine effectiveness estimates (VE-TND), the original RCT vaccine efficacy estimates (VE-RCT), and the relative percent difference between these estimates in the RV1 trial. During the first year of life (<1 years of age), the country-combined and country-specific efficacy and effectiveness estimates for all doses were similar (two or three doses combined in South Africa and Malawi: VE-TND: 58.2% [95%CI: 35.5–72.9]; VE-RCT: 61.2% [95%CI: 44.0–73.2]; −4.9% relative difference). The sub-cohort in South Africa followed over two rotavirus seasons yielded a low sample of rotavirus-positive cases resulting in less robust estimates during both the second year of life (1−<2 years of age) and using complete follow-up (<2 years of age). The sub-cohort in Malawi followed over two rotavirus seasons resulted in VE-TND and VE-RCT estimates which were not meaningfully different during any periods of follow-up.

VE-TND and VE-RCT estimates for the RV5 trial in sub-Saharan Africa are shown in Table 3. Using diarrheal episodes identified in the first year of life (<1 years of age), the VE-TND and the VE-RCT are almost identical, particularly in the country-combined estimate (VE-TND: 66.9% [95%CI: 42.7–80.9]; VE-RCT: 64.2% [95%CI: 40.2–79.4]; 4.2% relative difference). In the second year of life (1–<2 years of age) the VE-TND was greater than the VE-RCT for both country-combined and country-specific estimates in the second year (combined African study sites: VE-TND: 39.4% [95%CI: 5.0–61.4]; VE-RCT: 19.6% [95%CI: −15.7–44.4]; 101.0% relative difference). Using complete follow-up (<2 years of age), the VE-TND was moderately greater than the VE-RCT in all study settings (combined African study sites: VE-TND: 51.9% [95%CI: 32.1–65.9]; VE-RCT: 39.3% [95%CI: 19.1–54.7]; 32.1% relative difference).

In this trial, the rotavirus season in Ghana occurred between January and March. After restricting the analysis to diarrheal episodes during this time period (Table 3), there was no meaningful difference between the VE-TND and VE-RCT estimates (VE-TND: 56.1% [95%CI: −8.3–82.2]; VE-RCT: 55.5% [95%CI: 28.0–73.1]; 1% relative difference). Similarly, after restricting the analysis in Mali to the rotavirus season (October through February), the relative difference between VE-TND and VE-RCT estimates decreased (VE-TND: 9.4% [95%CI: −73.3–52.7]; VE-RCT: 17.6% [95%CI: −22.9–45.0]; −46.6% relative difference). In both rotavirus-season restricted analyses, sample size decreased substantially. Combining diarrheal episodes in Ghana and Mali during their respective rotavirus seasons with year-round diarrheal episodes in Kenya, resulted in a 5.3% relative difference between country-combined VE-TND and VE-RCT estimates.

Table 4 shows the VE-TND and VE-RCT estimates for the RV5 trial in Asia. VE-TND and VE-RCT estimates were not meaningfully different in country-combined analyses or during any periods of follow-up (combined Asian study sites using complete follow-up (<2 years of age): VE-TND: 49.8% [95%CI: 14.6–70.5]; VE-RCT: 48.3% [95%CI: 22.3–66.1]; 3.1% relative difference).

**Fig. 1:**
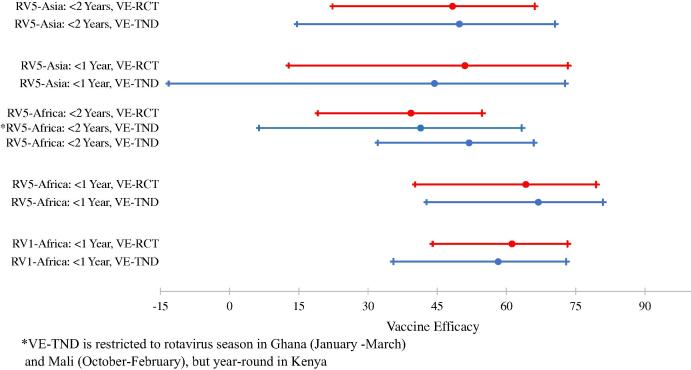
VE-TND and VE-RCT estimates and 95% confidence intervals for each rotavirus vaccine RCT.

Fig. 1 summarizes the VE-TND and VE-RCT estimates and overlapping 95% CIs for all RCTs during the first year of life and using the complete follow-up (<2 years of age).

Table 5 shows both RV1 and RV5 vaccine efficacy against severe rotavirus-negative diarrhea (VE-NEG). In the RV1 trial, the magnitude of VE-NEG was greatest in South Africa during the second year of follow-up with three doses of vaccine (54.0% [95%CI: 7.1–77.2]), while all other age and dose combinations had a low VE-NEG. In the RV5 trial in sub-Saharan Africa, the magnitude of the VE-NEG was greatest in Ghana during the second year of life (1–<2 years of age: −50.5 [95%CI: −170.9–14.9]), and using complete follow-up (<2 years of age: −49.1 [95%CI: −117.9 to −2.7]), compared to other settings. The country-combined VE-NEG was statistically significant using complete follow-up. After restricting the analysis to the rotavirus season using complete follow-up, the VE-NEG decreased in all RV5 settings with no statistically significant estimates. In the RV5 trial in Asia, the magnitudes of the country-combined VE-NEG estimates were low (<20%) during all follow-up periods. Due to the low magnitude of each VE-NEG, the ratio of vaccine: placebo test-negative controls replicated the 2:1 or 1:1 randomized vaccine coverage of the underlying study population in most study settings.

## 4 Discussion

Overall, the results from the TND analysis for RV1 and RV5 in sub-Saharan Africa and Asia were similar to primary efficacy results. The heterogeneity of vaccine effectiveness estimates between countries was also observed. In countries with a marked rotavirus season, estimates were not meaningfully different after restricting analyses to these time periods. We also demonstrated that RV1 and RV5 had no effect on severe rotavirus-negative diarrhea, a key assumption of the TND.

VE-TND during the first year of life was comparable to VE-RCT for all three trials. The control group accurately represented the vaccine coverage of the underlying study population. For example, in the RV1 trial the ratio of vaccinated (two doses) controls to placebo controls is nearly 1:1 (93:91), the randomized vaccine: placebo ratio. In all trials, VE-TND estimates during the second year of life were largely limited by the low number of diarrheal episodes and rotavirus-positive cases identified. Primary VE-RCTs for the second year of life were not statistically significant and this was replicated in the VE-TND results. The sustained effect of rotavirus vaccine during the second year of life remains unclear [3], [28].

In the analysis using complete follow-up (<2 years of age), the VE-TND was similar to the VE-RCT in RV1 and RV5-Asia. Differences between the estimates were demonstrated in the RV5 trial in sub-Saharan Africa for country-combined and country-specific estimates. Ghana and Mali have distinct rotavirus seasons. Analyses were restricted to the rotavirus season in order to obtain time-matched controls. This strategy better emulates the practice of incidence density sampling and provides results that are closer to the relative risk (and in turn VE = 1-Relative Risk) derived from an RCT. These results support the importance of temporally matched controls or accounting for timing of birth and case presentation in the TND to obtain accurate vaccine effectiveness estimates.

Generally, less robust VE-TND and VE-RCT estimates in Mali are in part due to changes in surveillance after the first year of the study, which initially missed most of the rotavirus season and yielded a low number of rotavirus cases. Surveillance and community engagement increased during the second year of the trial to increase case capture [5]. Notably, during the first year the ratio of vaccinated controls to placebo controls is almost exactly 1:1 (48:49), indicating the surveillance appropriately identified the underlying vaccine coverage in test-negative controls during this time.

Further evidence of the accuracy of the test-negative control group is demonstrated in rotavirus vaccine case-control studies using multiple control groups in sub-Saharan Africa and Latin America [17], [19], [20], [23], [29]. In Malawi, RV1 effectiveness for severe rotavirus-positive diarrhea during the first year of life was similar using test-negative controls and community controls (VE-TND: 68%, VE-Community controls: 68%) [17]. In South Africa, RV1 effectiveness for hospital admission with rotavirus-positive diarrhea in children <2 years old was comparable using test-negative controls and hospitalized controls (VE-TND: 57%, VE-Hospital controls: 63%) [20]. In Bolivia, RV1 effectiveness for severe rotavirus-positive diarrhea during the first year of life was moderately different using test-negative controls and hospitalized controls (VE-TND: 66%, VE-Hospital controls: 78%) [29]. In Nicaragua, RV5 effectiveness for severe rotavirus-positive diarrhea during the first year of life was slightly different using test-negative controls and a combined group of hospitalized and community controls (VE-TND: 70%, VE-Hospital/Community controls: 83%). In children >1 years old, the difference in estimates was more substantial (VE-TND: 33%, VE-Hospital/Community controls:70%) [19]. Authors suggest the combined control group differed from the test-negative and test-positive participants, likely due to healthcare seeking behaviors. In Guatemala RV1 and RV5 effectiveness against a rotavirus-positive diarrheal episode resulting in a hospital visit was moderately different using test-negative controls and hospitalized controls (VE-TND: 52%, VE-Hospital controls: 74%) [23]. All studies observed vaccine effectiveness results similar to efficacy estimates of RCTs conducted in Latin America.

Importantly, while the TND can be valuable, this study design is susceptible to biases present in all observational studies including selection bias, confounding, and misclassification of vaccine and rotavirus status [14]. In these RCTs we are highly confident of accurate vaccine ascertainment, but this can be problematic in the field with missing documentation, unreliable parental recall, and the added complexities of multiple doses [30]. Additionally, the sensitivity and specificity of the test to identify the etiologic pathogen is especially important when using the TND. A test with low specificity influences results more substantially than a test with low sensitivity [10]. All RCTs and case-control studies used ELISA for rotavirus detection. While RT-PCR is the gold standard, ELISA has high sensitivity (75–82%) and specificity (100%) to identify rotavirus [31]. A simulation study comparing true and estimated TND vaccine effectiveness results based on varying rotavirus test characteristics and attack rates demonstrated minimal bias with the currently used ELISA [32].

This analysis evaluated the use of the TND to estimate rotavirus vaccine effectiveness in low-income settings. Three separate rotavirus vaccine trials, testing two vaccines in seven countries, showed TND vaccine effectiveness estimates were nearly identical to the primary efficacy estimates of the original RCTs. The key assumption of the TND, the vaccine has no impact on rotavirus-negative diarrhea, was also upheld. This study supports the test-negative design as an appropriate method to measure rotavirus vaccine effectiveness in low-income settings.

## Funding

This publication is based on research funded in part by the Bill & Melinda Gates Foundation [OPP1068644] and the National Institutes of Health [NIH/NIAID MERIT R37 AI032042]. The findings and conclusions contained within are those of the authors and do not necessarily reflect positions or policies of the Bill & Melinda Gates Foundation or the National Institutes of Health. Funders of the study had no role in study design, data collection, data analysis, data interpretation, writing of the report, or the decision to submit the article for publication.

## Contributors

All authors contributed to the design of the study. LMS and JCV obtained the data. LMS analyzed the data. All authors interpreted the data. LMS wrote the manuscript and all authors revised the manuscript. All authors approved the final version of the manuscript.

## Conflict of interest

There are no conflicts of interest by the authors.

## Figures and Tables

**Table 1:** Summary of rotavirus vaccine clinical trials in low-income settings.

Vaccine	Dosing schedule	Surveillance type	Study site	Age during follow-up	Primary per-protocol population (Vaccine/Placebo)	Country specific per-protocol population (Vaccine/Placebo)	Reference
Rotarix (RV1)	6, 10, 14 weeks or 10,14 weeks	Active: Scheduled weekly home visits and clinic visits	South Africa	<1 Years	2974/1443	1944/960	[2]
			Malawi			1030/483	
			South Africa	1–<2 Years	⁎	686/332	[3]

Malawi	814/380	[4]

RotaTeq (RV5)	6, 10, 14 weeks	Passive: clinic visits	Ghana	<2 Years	2404/2385	940/930	[5]
			Kenya			573/577	
			Mali			891/878	
RotaTeq (RV5)	6, 10, 14 weeks	Passive: clinic visits	Bangladesh	<2 Years	995/988	557/561	[6]
			Vietnam			438/427	

⁎ Vaccine efficacy was estimated separately in South Africa and Malawi for the second year of this study.

**Table 2:** RV1 test-negative design results.

Study Site	Age (Years)	Doses	Cases⁎	Controls⁎	VE-TND (95%CI)		VE-RCT (95%CI)		% Relative difference⁎⁎
South Africa and Malawi	<1	2 or 3 doses	56/70	174/91	58.2	(35.5–72.9)	61.2	(44.0–73.2)	−4.9
		2 doses	30/70	93/91	58.1	(29.7–75.0)	58.7	(35.7–74.0)	−1.0
		3 doses	26/70	81/91	58.3	(28.3–75.7)	63.7	(42.4–77.8)	−8.5

South Africa	<1	2 or 3 doses	15/32	46/25	74.5	(44.3–88.4)	76.9	(56.0–88.4)	−3.1
		2 doses	9/32	24/25	70.7	(25.9–88.4)	72.2	(40.4–88.3)	−2.1
		3 doses	6/32	22/25	78.7	(39.5–92.5)	81.5	(55.1–93.7)	−3.4

Malawi	<1	2 or 3 doses	41/38	128/66	44.4	(5.3–67.3)	49.4	(19.2–68.3)	−10.1
		2 doses	21/38	69/66	47.1	(0.7–71.9)	49.2	(11.1–71.7)	−4.3
		3 doses	20/38	59/66	41.1	(−12.3–69.1)	49.7	(11.3–72.7)	−17.3

South Africa	1–<2	2 or 3 doses	5/4	32/23	10.2	(−271.6–78.3)	40.0	(−204.0–87.0)	−74.5
		2 doses	4/4	21/23	−9.5	(−394.2–75.7)	3.0	(−43.0–82.0)	−416.7
		3 doses	1/4	11/23	47.4	(−424.6–94.8)	76.0	(−143.0–100.0)	−37.6

Malawi	1–<2	2 or 3 doses	30/17	89/45	10.8	(−78.7–55.5)	17.6	(−59.2–56.0)	−38.6
		2 doses	18/17	44/45	−8.3	(−136–50.5)	2.6	(−101.2–52.6)	−419.2
		3 doses	12/17	45/45	29.4	(−64.6–68.7)	33.1	(−48.6–70.9)	−11.2

South Africa	<2	2 or 3 doses	11/13	58/32	53.3	(−16.2–81.2)	59.0	(1.0–83.0)	−9.7
		2 doses	9/13	38/32	41.7	(−54.0–77.9)	32.0	(−71.0–75.0)	30.3
		3 doses	2/13	20/32	75.4	(−20.7–95.0)	85.0	(35.0–98.0)	−11.3

Malawi	<2	2 or 3 doses	69/53	183/90	36.0	(0.8–58.7)	38.1	(9.8–57.3)	−5.5
		2 doses	38/53	96/90	32.8	(−11.5–59.5)	34.0	(−2.0–57.7)	−3.5
		3 doses	31/53	87/90	39.5	(−3.0–64.5)	42.3	(8.8–64.0)	−6.6

Cases: severe (Vesikari ⩾ 11) rotavirus-positive diarrhea.

Controls: severe rotavirus-negative diarrhea.

VE-TND: Vaccine effectiveness against severe rotavirus diarrhea using the test-negative design.

VE-RCT: Vaccine efficacy against severe rotavirus diarrhea-original randomized control trial estimates.

⁎ No. Vaccine/No. Placebo.

⁎⁎ VE-TND compared to VE-RCT.

**Table 3:** RV5 test-negative design results in sub-Saharan Africa.

Study Site	Age (Years)	Cases⁎	Controls⁎	VE-TND (95%CI)		VE-RCT (95%CI)		% Relative difference⁎⁎
African study sites	<1	23/61	115/101	66.9	(42.7–80.9)	64.2	(40.2–79.4)	4.2
Ghana		16/42	44/36	68.8	(35.6–84.9)	65.0	(35.5–81.9)	5.8
Kenya		2/14	23/16	90.1	(50.1–98.0)	83.4	(25.5–m98.2)	8.0
Mali		5/5	48/49	−2.1	(−275.3–72.2)	1.0	(−431.7–81.6)	−310.0

African study sites	1–<2	57/71	110/83	39.4	(5.0–61.4)	19.6	(−15.7–44.4)	101.0
Ghana		11/15	33/22	51.1	(−26.0–81.0)	29.4	(−64.6–70.7)	73.8
Kenya		3/2	8/11	−106.3	(−1435.7–72.3)	−54.7	(−1752.7–82.3)	94.3
Mali		43/54	69/50	42.3	(0.9–66.4)	19.2	(−23.1–47.3)	120.3

African study sites	<2	80/132	208/165	51.9	(32.1–65.9)	39.3	(19.1–54.7)	32.1
Ghana		27/57	74/50	68.0	(42.7–82.1)	55.5	(28.0–73.1)	22.5
Kenya		5/16	27/25	71.1	(9.3–90.8)	63.9	(−5.9–89.8)	11.3
Mali		48/59	107/90	31.6	(−9.8–57.4)	17.6	(−22.9–45.0)	79.5

*Restricted to Rotavirus Season*								
African study sites^a^	<2	69/123	68/71	41.4	(6.4–63.3)	39.3	(19.1–54.7)	5.3
Ghana^a^		22/54	13/14	56.1	(−8.3–82.2)	55.5	(28.0–73.1)	1.1
Kenya		5/16	27/25	71.1	(9.3–90.8)	63.9	(−5.9–89.8)	11.3
Mali^a^		42/53	28/32	9.4	(−73.3–52.7)	17.6	(−22.9–45.0)	−46.6

Cases: severe (Vesikari ⩾ 11) rotavirus-positive diarrhea.

Controls: severe rotavirus-negative diarrhea.

VE-TND: Vaccine effectiveness against severe rotavirus diarrhea using the test-negative design.

VE-RCT: Vaccine efficacy against severe rotavirus diarrhea-original randomized control trial estimates.

⁎ No. Vaccine/No. Placebo.

⁎⁎ VE-TND compared to VE-RCT.

a Cases/controls restricted to rotavirus season in Ghana (January-March) and Mali (October-February), but year-round in Kenya.

**Table 4:** RV5 test-negative design results in Asia.

study site	Age (Years)	Cases⁎	Controls⁎	VE-TND (95%CI)		VE-RCT (95%CI)		% Relative difference⁎⁎
Asian study sites	<1	19/38	36/40	44.4	(−13.2–72.7)	51.0	(12.8–73.3)	−12.9
Bangladesh		17/31	31/38	32.8	(−43.5–68.5)	45.7	(−1.2–71.8)	−28.2
Vietnam		2/7	5/2	88.6	(−10.8–98.8)	72.3	(−45.2–97.2)	22.5

Asian study sites	1–<2	19/34	27/23	52.4	(−4.9–78.4)	45.5	(1.2–70.7)	15.2
Bangladesh		16/25	23/18	42.3	(0.9–66.4)	39.3	(−18.3–69.7)	7.6
Vietnam		3/9	4/5	58.3	(−166.0–93.5)	64.6	(−47.7–93.9)	−9.8

Asian study sites	<2	38/72	62/59	49.8	(14.6–70.5)	48.3	(22.3–66.1)	3.1
Bangladesh		33/56	53/52	42.2	(−2.8–67.5)	42.7	(10.4–63.9)	−1.2
Vietnam		5/16	9/7	75.7	(0.6–94.1)	63.9	(7.6–90.9)	18.5

Cases: severe (Vesikari ⩾ 11) rotavirus-positive diarrhea.

Controls: severe rotavirus-negative diarrhea.

VE-TND: Vaccine effectiveness against severe rotavirus diarrhea using the test-negative design.

VE-RCT: Vaccine efficacy against severe rotavirus diarrhea-original randomized control trial estimates.

⁎ No. Vaccine/No. Placebo.

⁎⁎ VE-TND compared to VE-RCT.

**Table 5:** RV1 and RV5 vaccine efficacy against severe rotavirus-negative diarrhea results.

Vaccine	Study site	Age (Years)	Doses	VE-NEG (95%CI)	
RV1	South Africa and Malawi	<1	2 or 3 doses	7.2	(−18.6–27.4)
			2 doses	1.4	(−30.4–25.5)
			3 doses	13.1	(−16.2–35.0)

	South Africa	<1	2 or 3 doses	9.1	(−47.0–43.8)
			2 doses	5.1	(−65.0–45.4)
			3 doses	13.2	(−52.9–50.7)

	Malawi	<1	2 or 3 doses	9.1	(−19.9–31.0)
			2 doses	3.8	(−31.7–29.7)
			3 doses	14.5	(−18.7–38.4)

	South Africa	1–e<2	2 or 3 doses	32.7	(−13.2–60.0)
			2 doses	11.1	(−57.5–49.8)
			3 doses	54.0	(7.1–77.2)

	Malawi	1–<2	2 or 3 doses	7.7	(−29.4–34.1)
			2 doses	10.0	(−33.1–39.2)
			3 doses	5.2	(−39.8–35.8)

	South Africa	<2	2 or 3 doses	12.3	(−32.9–42.1)
2 doses			−15.9	(−81.8–26.1)
3 doses			40.0	(−3.2–65.1)

Malawi	<2	2 or 3 doses	4.7	(−19.8–24.1)
		2 doses	1.9	(−27.2–24.3)
		3 doses	7.5	(−20.7–29.2)

RV5	African study sites	<1	3 doses	−13.3	(−49.6–14.0)
	Ghana			−21.4	(−94.1–23.6)
	Kenya			−46.4	(−196.6–25.9)
	Mali			3.7	(−46.5–36.7)

	African study sites	1–<2	3 dosesm	−33.1	(−79.1–0.8)
	Ghana			−50.5	(−170.9–14.9)
	Kenya			27.3	(−98.5–74.6)
	Mali			−38.9	(−104.1–4.9)

	African study sites	<2	3 doses	−26.5	(−56.1 to −2.6)
	Ghana			−49.1	(−117.9 to −2.7)
Kenya	−9.2			(−96.1–39.0)
Mali	−18.4			(−58.5–11.4)

RV5: *Restricted to Rotavirus Season*^a^	African study sites	<2	3 doses	5.1	(−34.3–32.9)
	Ghana			8.1	(−110.7–60.2)
	Kenya			−9.2	(−96.1–39.0)
	Mali			−13.9	(−47.5–50.1)

RV5	Asian Study Sites	<1	3 doses	10.8	(−43.6–44.7)
	Bangladesh			18.5	(−34.6–50.9)
	Vietnam			−145.3	(−2375.8–59.8)

	Asian Study Sites	1–<2	3 doses	−17.2	(−113.9–35.3)
	Bangladesh			−38.9	(−104.1–4.9)
	Vietnam			22.0	(−262.4–84.5)

	Asian Study Sites	<2	3 doses	−4.8	(−52.3–27.8)
Bangladesh	−2.8			(−53.7–31.2)
Vietnam	−26.0			(−298.3–58.2)

a Cases/controls restricted to rotavirus season in Ghana (January-March) and Mali (October-February), but year-round in Kenya.
